# Generation of magnetic biohybrid microrobots based on MSC.sTRAIL for targeted stem cell delivery and treatment of cancer

**DOI:** 10.1186/s12645-023-00203-9

**Published:** 2023-05-19

**Authors:** Rebekah Anamarie Gundersen, Tianyuan Chu, Kiana Abolfathi, Serap Gokcen Dogan, Phoebe Elizabeth Blair, Nyasha Nago, Michael Hamblin, Greg Nicholas Brooke, Ralf Michael Zwacka, Ali Kafash Hoshiar, Andrea Mohr

**Affiliations:** 1 School of Life Sciences, Protein Structure and Mechanism of Disease Group, Cancer and Stem Cell Biology Laboratory, University of Essex, Colchester CO4 3SQ, UK; 2 School of Computer Science and Electronic Engineering, University of Essex, Colchester CO4 3SQ, UK; 3 Haematology Unit, East Suffolk and North Essex NHS Foundation Trust, Colchester CO4 5JL, UK; 4 School of Life Sciences, Protein Structure and Mechanism of Disease Group, Molecular Oncology Laboratory, University of Essex, Colchester CO4 3SQ, UK

**Keywords:** Biohybrid microrobots, Mesenchymal stromal cells, sTRAIL, sPD1HAC, Rotating magnetic field, Mesenchymal stem cells, Cancer

## Abstract

**Background:**

Combining the power of magnetic guidance and the biological activities of stem cells transformed into biohybrid microrobots holds great promise for the treatment of several diseases including cancer.

**Results:**

We found that human MSCs can be readily loaded with magnetic particles and that the resulting biohybrid microrobots could be guided by a rotating magnetic field. Rotating magnetic fields have the potential to be applied in the human setting and steer therapeutic stem cells to the desired sites of action in the body. We could demonstrate that the required loading of magnetic particles into stem cells is compatible with their biological activities. We examined this issue with a particular focus on the expression and functionality of therapeutic genes inside of human MSC-based biohybrid microrobots. The loading with magnetic particles did not cause a loss of viability or apoptosis in the human MSCs nor did it impact on the therapeutic gene expression from the cells. Furthermore, the therapeutic effect of the gene products was not affected, and the cells also did not lose their migration potential.

**Conclusion:**

These results demonstrate that the fabrication of guidable MSC-based biohybrid microrobots is compatible with their biological and therapeutic functions. Thus, MSC-based biohybrid microrobots represent a novel way of delivering gene therapies to tumours as well as in the context of other diseases.

## Background

Mesenchymal stromal cells (also known as mesenchymal stem cells; MSCs) are of particular interest as cellular delivery vehicles in several treatment areas including tumour diseases (Mohr and Zwacka 2018; Lan et al. 2021). Their use in anti-cancer therapies is informed by their inherent tumour tropism that is based on their normal function in wound healing processes. In this context tumours are sensed by MSCs as a “wound that never heals” (Dvorak 2015, 1986; Mohr and Zwacka 2018). When used in anti-cancer therapies, MSCs are usually genetically modified ex vivo to carry a gene that affords their therapeutic effect. ‘The gene products can have either a direct impact on the tumour like causing cancer cell death or trigger a more systemic mechanism such as activation of the immune system to tackle the cancer (Mohr et al. 2008; Studeny et al. 2004). Alternatively, MSCs can be administered to deliver chemotherapeutic drugs to tumours (Szewc et al. 2022). Generally, MSCs offer better and more specific delivery of therapeutic agents than the systemic administration of high-dose cytotoxic drugs or biologicals where the bioavailability at the site of tumour growth is often insufficient (de Miguel et al. 2016).

In preclinical studies we have demonstrated that MSC-delivery of an engineered soluble tumour necrosis factor-related apoptosis-inducing ligand (sTRAIL) has very significant anti-cancer effects in multiple cancer types (Mohr et al. 2010, 2019, 2008; Yu et al. 2013). TRAIL is a member of the TNF superfamily and is normally a type II transmembrane protein that can rapidly induce programmed cell death (known as apoptosis) in cancer cells but not in normal cells (Ashkenazi et al. 1999). TRAIL binds to a receptor system consisting of two apoptosis-inducing agonistic death receptors, death receptor 4 (DR4/TRAIL-R1) and death receptor 5 (DR5/TRAIL-R2), and three antagonistic or decoy receptors, decoy receptor 1 (DcR1/TRAIL-R3), decoy receptor 2 (DcR2/TRAIL-R4), and the soluble receptor osteoprotegerin (OPG) (von Karstedt et al. 2017).

TRAIL binds as a homotrimer to its receptors, which in turn also trimerise, and in the case of TRAIL-R1 and TRAIL-R2 leads to the recruitment of Fas-associated death domain (FADD). FADD then mediates the binding and activation of caspase-8. Activated caspase-8 activates downstream effector caspases such as caspase-3, which then execute apoptosis. The three decoy receptors cannot induce cell death (Micheau et al. 2013). We engineered a version of TRAIL that is no longer membrane bound and can be expressed and secreted by MSCs (Mohr et al. 2010). This form of TRAIL was named sTRAIL and is based on the extracellular domain of TRAIL. Furthermore, it was rendered hyperpotent by forcing the trimerisation of the produced TRAIL molecules via the insertion of an isoleucine zipper in our synthetic TRAIL gene (Kim et al. 2008; Tuthill et al. 2015).

In our preclinical studies, sTRAIL produced and delivered by MSCs achieved better apoptosis-inducing activity than conventional full-length and membrane-bound TRAIL (Mohr et al. 2010, 2019; Yu et al. 2013). Despite the success of these studies, the number of MSCs found in the primary tumours was relatively low (Albarenque et al. 2011). Therefore, an approach that directs more therapeutic MSCs to tumour tissues promises significant benefits. A strategy to improve the tumour targeting of MSCs is to transform them into biohybrid microrobots (BHM-MSCs). For this, MSCs are loaded with super-paramagnetic iron oxide nanoparticles (SPIONs) and steered by magnetic forces. As part of cancer therapies, the BHM-MSCs can be guided to the cancerous lesion and thereby enriched in the tumour tissue. Not being able to detect and visualise single microrobots in vivo makes it difficult to control them (Yu et al. 2019). Therefore, grouping and guiding them as a microswarm that can be readily detected with conventional bioimaging technologies has been proposed as a possible solution (Yu et al. 2018). This can be achieved with different accentuation schemes (Abolfathi et al. 2020; Liu et al. 2010; Muthana et al. 2015; Park et al. 2020). Initial works on developing microswarms were centered on gradient based magnetic steering (Amin et al. 2017; Hoshiar et al. 2017). Such gradient based steering, however, required a very high gradient field (> 2 T/m) which limited its implementation. To overcome this hurdle, actuation by a rotating magnetic field emerged (Yang et al. 2022; Wang et al. 2021).

SPIONs, from thereon referred to as magnetic particles (MPs), have been shown to have no influence on the characteristics of a variety of different cell types such as human nasal turbinate stem cells (Jeon et al. 2021), macrophages (Han et al. 2016) or MSCs (Harrison et al. 2017; Yun et al. 2018). The biohybrid microrobots were also still capable of proliferation and differentiation (Harrison et al. 2017; Jeon et al. 2021). In the present study, we generated BHM-MSCs expressing and secreting different therapeutic transgenes. We found that MP uptake does not affect the survival, magnetic guidance and therapeutic transgene expression and secretion from MSCs. Furthermore, the conversion of MSCs into biohybrid microrobots did not affect their migratory characteristics and thereby increases their efficacy.

## Results

### Loading of MSCs with MPs

**Fig. 1:**
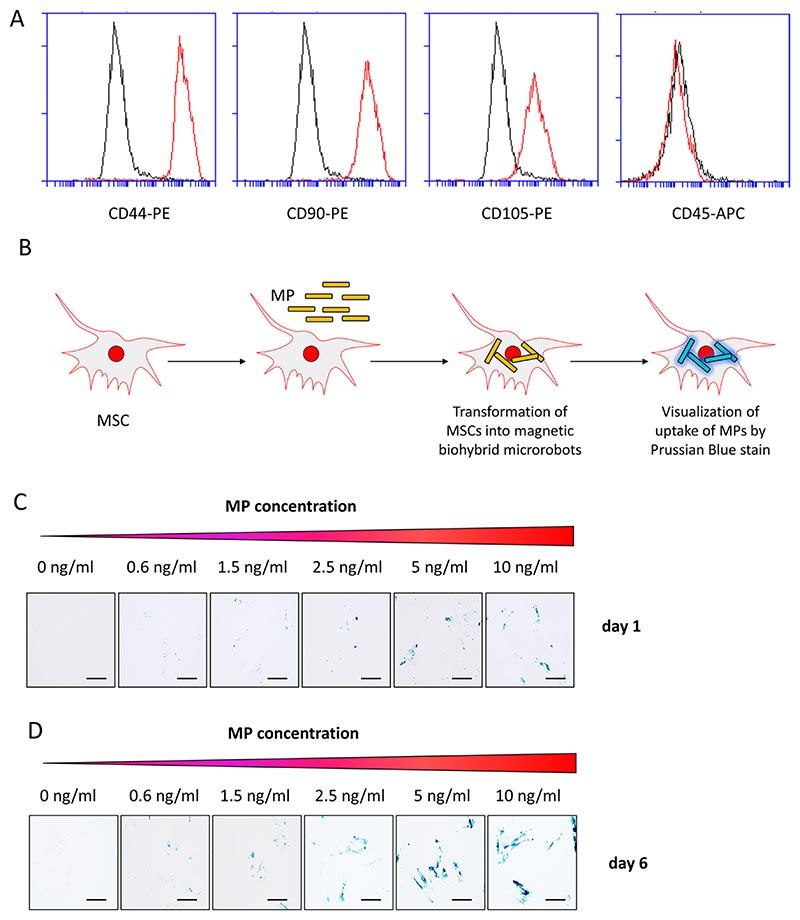
Generation of BHM-MSCs. **A** Surface marker stain of MSCs. FACS analyses of human MSCs for surface expression of CD44, CD90, CD105, and CD45. Their specific signals are shown in red, with respective isotype controls in black. **B** Schematic overview of the experimental procedure of BHM-MSC generation. **C** Prussian blue staining of human MSCs with increasing concentrations of MPs (0 ng/ml, 0.6 ng/ml, 1.5 ng/ml, 2.5 ng/ml, 5 ng/ml, and 10 ng/ml). Blue colouring indicates ferric iron particles after 1 day. Scale bars = 125 μm. **D** Prussian blue staining of human MSCs with increasing concentrations of MPs (0 ng/ml, 0.6 ng/ml, 1.5 ng/ml, 2.5 ng/ml, 5 ng/ml, and 10 ng/ml). Blue colouring indicates ferric iron particles after 6 days. Scale bars = 125 μm

First, we examined the human MSCs we used to generate BHM-MSCs for their stem cell character by surface marker expression analyses. The results revealed that the MSCs were positive for CD44, CD90 and CD105 while being negative for CD45 (Fig. 1a), corresponding to a typical MSC profile (Dominici et al. 2006). In order to engineer human MSCs into BHM-MSCs that can be guided by rotating magnetic fields to specific target tissues like tumours, we evaluated their capacity to be loaded with 1 μm MPs (Fig. 1b). For this, we incubated MSCs with varying concentrations (0.6–10 ng/ml) of MPs and confirmed the particle uptake by Prussian blue staining after 1 and 6 days, respectively. We detected a rise in the uptake of particles inside of MSCs with increasing concentrations on day 1 (Fig. 1c). This increase was even more prominent after 6 days (Fig. 1d) and demonstrates particle retention inside of the MSCs for at least 1 week.

### Guidance of BHM-MSCs by rotating magnetic fields

**Fig. 2:**
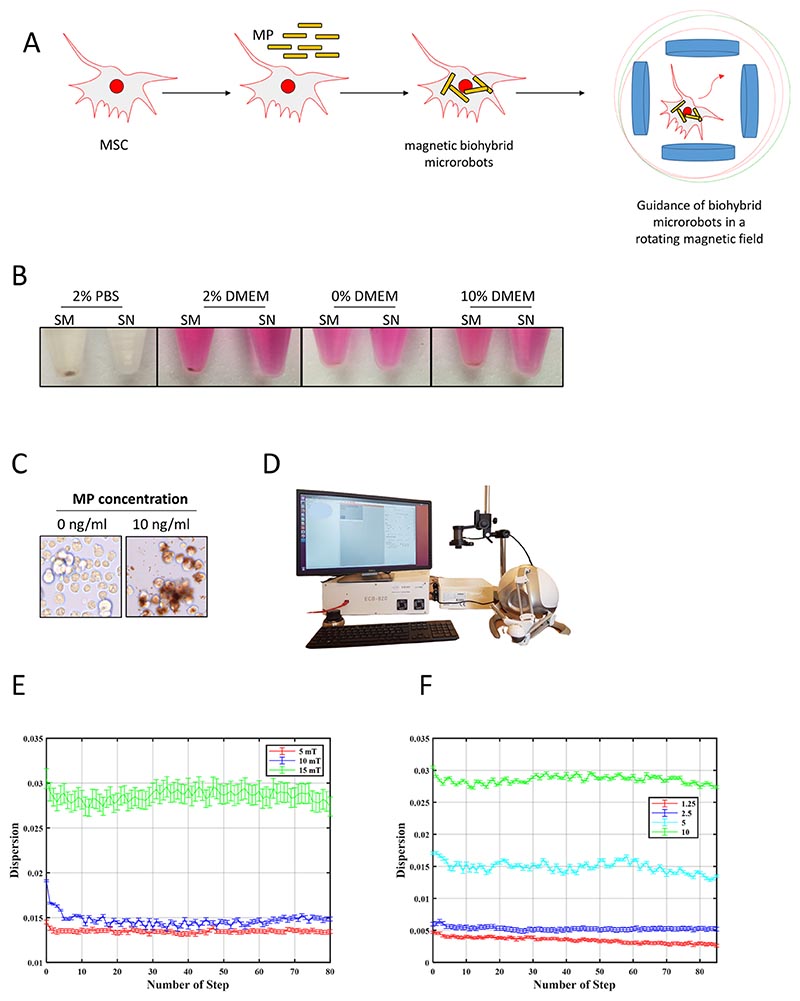
Guidance of BHM-MSCs in rotating magnetic fields. **A** Schematic overview of the generation of BHM-MSCs for magnetic guidance. **B** Pictures of MPs diluted in PBS supplemented with 2% FBS or DMEM with 0%, 2% or 10% FBS, respectively, and left for 24 h under cell culturing conditions. Of these samples 2 ml of the supernatants (SN) were transferred to a fresh tube. The remaining 2 ml were labelled as sediment (SM). **C** Brightfield images of MSCs (left) or BHM-MSCs (right) loaded with 10 ng/ml MPs. **D** The MFG100 magnetic micro-robotics system is employed to control the movement of the BHM-MSCs in rotating magnetic fields. The system is equipped with an optical camera (Basler, puA1280-54um) for real-time monitoring. **E** Dispersion of BHM-MSCs under three different magnetic fields, and the results demonstrate that higher magnetic fields lead to greater dispersions. The diagram displays the average outcomes of n = 3 videos. **F** Dispersion under four different MP concentrations (1.25 ng/ml, 2.5 ng/ml, 5 ng/ml and 10 ng/ml). It is noteworthy that, under comparable magnetic field conditions (frequency and field intensity), higher concentrations result in greater dispersions (n = 3 experiments)

Next, we set out to determine the optimal conditions for cellular uptake of MPs and magnetic guidance (Fig. 2a). First, we identified the medium compositions with the lowest MP agglomerations (Fig. 2b, Additional file 1: Fig. S1). This was found to be serum-free medium and this condition was used in all following experiments, as it also gave the best MP-loading results. Subsequently, we harvested the cells and confirmed MP uptake by microscopy (Fig. 2c), followed by exposure of the BHM-MSCs to rotating magnetic fields using an MFG100 system (Fig. 2d). Different intensities (< 15 mT) were applied to steer the microrobots. Video imaging clearly demonstrated control over movement and direction of the BHM-MSCs (Additional file 5). Furthermore, to describe the motion of BHM-MSCs, we introduced the concept of dispersion. In this context, dispersion refers to the extent to which BHM-MSCs are spread out within the magnetic field. A consistent dispersion can be used as a measure of the control exerted by rotating magnetic fields. For the calculation of dispersion, we converted videos depicting the movement of BHM-MSCs into images (Additional file 5). Then the MATLAB image processing toolbox (Release 2022, the MathWorks, USA) was used to extract geometrical data. A detailed explanation of dispersion for the swarm can be found in the reports by Abolfathi et al. (Abolfathi et al. 2020, 2022). Dispersion is a dimensionless parameter that quantifies the scattering of BHM-MSCs and is defined as follows:

(1) $$d=\frac{\sum_{i=1}^{N}r_{i}}{\sqrt{x_{c}^{2}+y_{c}^{2}}}$$

where the index *i* denotes the i^th^ BHM-MSC in the image, and *N* is the total number of BHM-MSCs in the image. The variables *x_c_* and *y_c_* represent the position of the center point of each image. The variable *r_i_* is the distance between the i^th^ BHM-MSC and the center point of the image, and is calculated as follows:

(2) $$r_{i}=\sqrt{{\left(x_{i}-x_{c}\right)}^{2}+{\left(y_{i}-y_{c}\right)}^{2}}$$

Figure 2e demonstrates the impact of magnetic field strength on the concentration of BHM-MSCs in a specific region when the frequency is set to 10 Hz. An incremental increase in the BHM-MSCs swarm concentration was observed for three different field intensities (5 mT, 10 mT, and 15 mT), and the pattern remained consistent over time. In addition, the effect of different concentrations of MPs used to generate BHM-MSCs was studied (Fig. 2f). The results indicate that higher concentrations lead to greater dispersion, and this trend remained constant. Taken together, these results mean that control could be imposed on the BHM-MSCs under the described experimental conditions.

### Biocompatibility of MPs and human MSCs

**Fig. 3:**
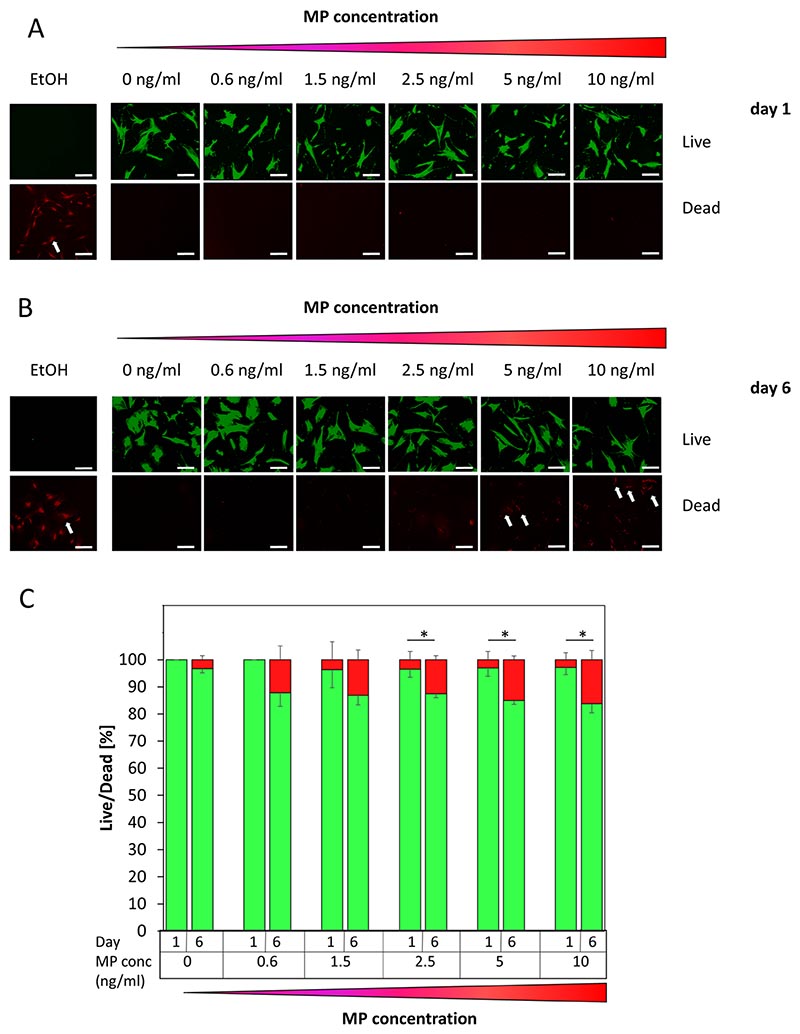
Fluorescent microscopic analysis of the effects of MPs on MSC viability over time. **A** Live/dead stain of MSCs incubated with increasing concentrations of MPs (0 ng/ml, 0.6 ng/ml, 1.5 ng/ml, 2.5 ng/ml, 5 ng/ml, and 10 ng/ml) for 1 day. Dead cells are indicated by a white arrow. Ethanol (EtOH) treated cells served as control. Scale bars = 125 μm. **B** Live/dead stain of MSCs incubated with increasing concentrations of MPs (0 ng/ml, 0.6 ng/ml, 1.5 ng/ml, 2.5 ng/ml, 5 ng/ml, and 10 ng/ml) for 6 days. Dead cells are pointed out by a white arrow. Ethanol (EtOH) treated cells served as control. Scale bars = 125 μm. **C** Quantification of percentage of dead cells (red) and live cells (green) compared between day 1 and day 6 across all concentrations of MPs in MSCs. Data are plotted as mean ± SEM. For the quantification a total of 15 random fields from each sample were counted

In the first biocompatibility analysis human MSCs were exposed to MPs at different concentrations ranging from 0.6 to 10 ng/ml, as this range represents concentrations at which BHM-MSCs could be guided. After 24 h a live/dead assay was carried out and the results revealed that very little cell death was caused by the MPs (Fig. 3a). When the MPs were left on the MSCs for 6 days the levels of cell death increased but were still relatively low with most cells still alive (Fig. 3b). Quantitative analyses showed that even at higher MP concentrations, no more than 5% cell death could be measured after 24 h (Fig. 3c). After 6 days cell death reached not more than around 15% in samples exposed to the highest particle concentration. Untreated cells showed around 3% of dead cells at this time point. These results demonstrate that MPs are well tolerated up to a period of 6 days and that the procedure of loading MSCs with MPs is likely to be compatible with their biological activity. The fact that more than 80% of MSCs are still alive after 6 days at the highest MP concentration provides a time window for them to be genetically modified ex vivo, administered, guided to tumour lesions and to effectively deliver their therapeutic anti-cancer payload as BHMs.

### MPs do not impact on therapeutic transgene expression

**Fig. 4:**
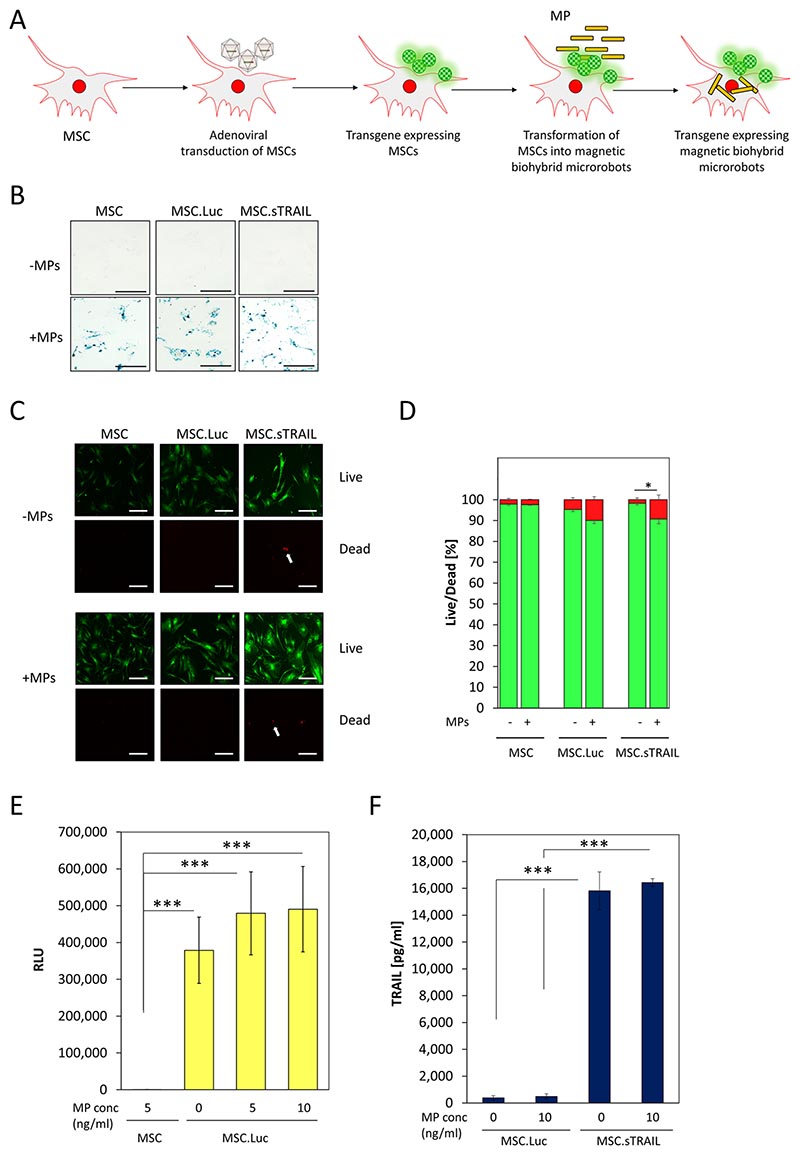
Transforming genetically modified MSCs into BHM-MSCs does not affect their transgene expression. **A** Schematic overview of the experimental procedure to generate transgene expressing BHM-MSCs. **B** Prussian blue stain of MSCs transduced with Ad.Luc (MSC.Luc) or Ad.sTRAIL (MSC.sTRAIL) with MP concentrations of 0 ng/ml (– MPs) and 10 ng/ml (+ MPs). Blue staining indicates presence of ferric iron particles. Scale bars = 250 μm. **C** Live/dead stain of untransduced MSCs (MSC), MSCs transduced with adenoviral vectors expressing luciferase (MSC.Luc) or sTRAIL (MSC.sTRAIL), with (+ MPs) or without MPs (– MPs). Live cells display green fluorescence, dead cells display red fluorescence. Examples of dead cells are indicated by a white arrow. Scale bars = 125 μm. **D** Quantification of percentage of dead cells (red) and live cells (green). Data are plotted as mean ± SEM. For the quantification a total of 15 random fields from each sample were counted. **E** Luciferase reporter assay of MSCs loaded with MPs at different concentrations and transduced with an adenoviral vector expressing luciferase (MSC.Luc). Luciferase activity is expressed as relative light units (RLU). Untransduced MSCs loaded with 5 ng/ml MPs were used as negative controls in the luciferase activity assay. Results depicted are from three independent experiments and plotted as mean ± SEM. **F** TRAIL expression and secretion was measured by ELISA in supernatants from MSCs transduced with adenoviral vectors expressing luciferase (MSC.Luc) or sTRAIL (MSC.sTRAIL), with or without MPs. Results depicted are from three independent experiments and plotted as mean ± SEM

After having shown that MPs do not affect survival of the BHM-MSCs, we tested whether (therapeutic) transgene expression is affected by loading with MPs or vice versa. MSCs were transduced with adenoviral vectors, as described previously (Mohr et al. 2010), either expressing Luciferase (Ad.Luc), sTRAIL (Ad.sTRAIL) or sPD1HAC (Ad.sPD1HAC). The resulting cells were named MSC.Luc, MSC.sTRAIL and MSC. sPD1HAC, respectively (Fig. 4a, Additional file 2: Fig. S2). Following transduction with adenoviral vectors, the MSCs were loaded with MPs at a concentration of 10 ng/ml before they were stained using the Prussian Blue method (Fig. 4b). The results show that MSCs genetically engineered by adenoviral transduction can be readily loaded with MPs showing no difference to non-transduced control cells. Furthermore, cell death was only very marginally increased after viral transduction and loading with MPs (Fig. 4c), never exceeding 10% after 3 days (Fig. 4d). Thus, the use of adenoviral vectors to modify MSCs is compatible with MP-loading and can be used in combination to modify MSCs to BHM-MSCs.

In addition, we quantified the transgene expression following adenoviral transduction and loading with MPs. When we used MSC.Luc with Luciferase expressed as a reporter gene, there was no decrease in expression of the enzyme after loading with either 5 or 10 ng/ml MPs (Fig. 4e). Next, we tested MSC.sTRAIL and MSC.sPD1HAC for expression and secretion of the respective soluble proteins. After the generation of BHM-MSCs from MSC.sTRAIL (BHM-MSC.sTRAIL) and MSC.sPD1HAC (BHM-MSC.sPD1HAC), sTRAIL and sPD1HAC protein levels were measured by ELISA in the supernatants of the cells. The results show that the MPs did not have an impact on the levels of the two therapeutic proteins (Fig. 4f and Additional file 2: Fig. S2) demonstrating that adenoviral transduction can be used to produce BHM-MSCs.

### BHM-MSCs-produced therapeutic transgenes maintain their therapeutic activity after loading with MPs

**Fig. 5:**
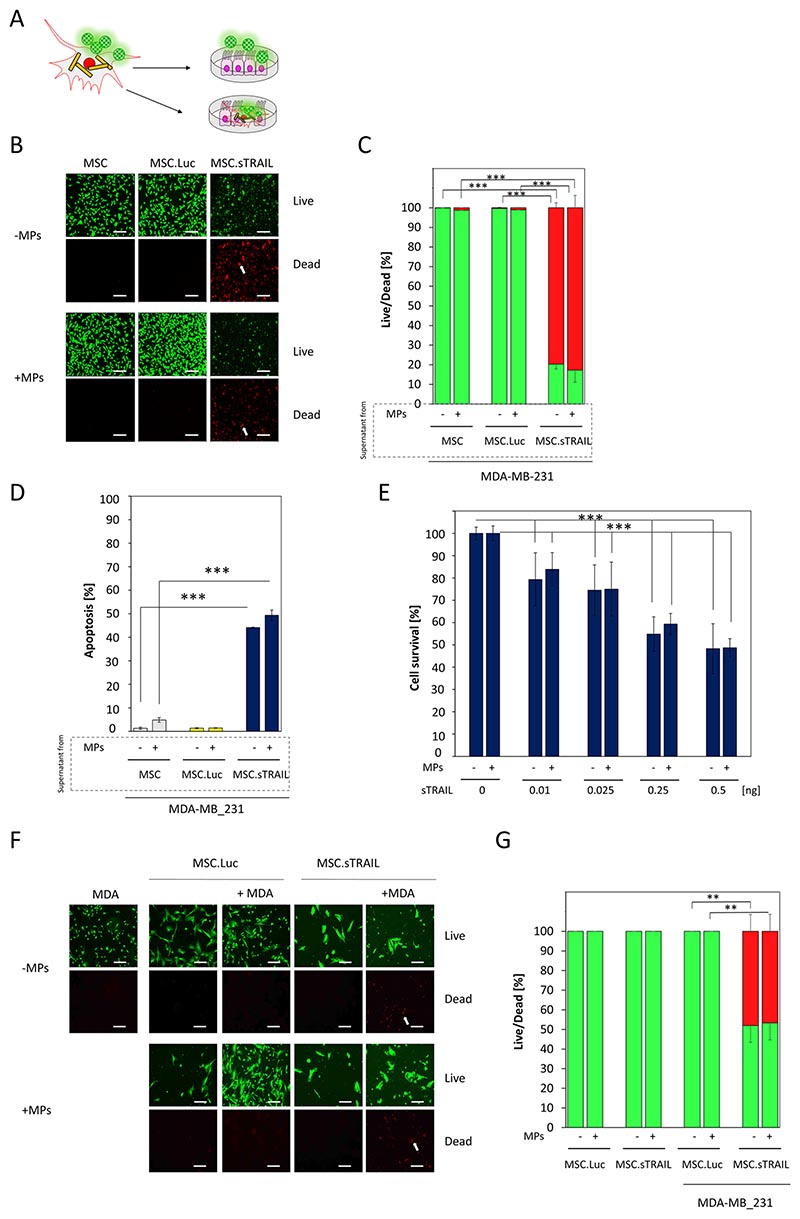
The sTRAIL secreted by BHM-MSCs is functional. **A** Schematic overview of the experimental design to test for sTRAIL functionality. In the first approach, supernatant from transgene expressing BHM-MSCs is transferred onto cancer cells. In the second approach, transgene expressing BHM-MSCs are directly mixed with cancer cells. **B** Live/dead stain of MDA-MB-231 breast cancer cells treated with supernatants from MSCs transduced with an adenoviral vector expressing sTRAIL (MSC.sTRAIL) with (+ MPs) or without MPs (– MPs). Live cells display green fluorescence, dead cells display red fluorescence. Examples of dead cells are indicated by a white arrow. Scale bars = 125 μm. **C** Quantification of percentage of dead MDA-MB-231 breast cancer cells (red) and live MDA-MB-231cells (green). Data are plotted as mean ± SEM. For the quantification a total of 15 random fields from each sample were counted. **D** MDA-MB-231 breast cancer cells treated with supernatants from untransduced MSCs (MSC), MSCs transduced with adenoviral vectors expressing luciferase (MSC.Luc) or sTRAIL (MSC.sTRAIL), with (+ MPs) or without MPs (– MPs). Apoptosis was measured by DNA-hypodiploidy assay and flow cytometry. Samples were measured in triplicate. Data are plotted as mean ± SEM. **E** MDA-MB-231 breast cancer cells treated with different concentrations of sTRAIL secreted from BHM-MSCs. After 7 days, cell survival was assessed by crystal violet staining. The measurement values of untreated cells were set to 100. Results are from three independent experiments. Data are plotted as mean ± SEM. (n = 3). **F** MDA-MB-231 breast cancer cells (MDA) mixed with MSCs transduced with adenoviral vectors expressing luciferase (MSC.Luc) or sTRAIL (MSC.sTRAIL), with (+ MPs) or without MPs (– MPs). Cell death was analysed after 3 days by live/dead staining. Live cells display green fluorescence, dead cells display red fluorescence. Dead cells are indicated by a white arrow. Scale bars = 125 μm. **G** Quantification of percentage of dead cells (red) and live cells (green). Data are plotted as mean ± SEM. For the quantification a total of 15 random fields from each sample were counted

Having demonstrated that the levels of therapeutic proteins are not affected in BHM-MSCs, we next examined whether the actual therapeutic activity was also not compromised by the loading with MPs (Fig. 5a). For this we used the supernatants of genetically modified MSCs as well as their biohybrid microrobotic counterparts and transferred them onto MDA-MB-231 breast cancer cells. A live/dead assay revealed that the supernatants of untransduced MSCs, MSC.Luc and BHM-MSC.Luc did not give rise to cell death in MDA-MB-231 cells, whereas the supernatants of MSC.sTRAIL caused substantial effects (Fig. 5b). The cell death-inducing activities were similar between MSC.sTRAIL and BHM-MSC.sTRAIL (Fig. 5b). Quantification of cell death showed that around 80% of the MDA-MB-231 cells were dead after exposure to supernatants from MSC.sTRAIL and BHM-MSC.sTRAIL, respectively (Fig. 5c). These cell death results were then corroborated with a more specific apoptosis assay. The employed DNA hypodiploidy test, which usually detects lower cell death levels than broader assays, like the live/dead stain, revealed that both MSC.sTRAIL and BHM-MSC.sTRAIL led to between 45 and 50% apoptosis in MDA-MB-231-cells (Fig. 5d). Similar results were obtained when we used even smaller concentrations of sTRAIL utilising a third independent assay. In this crystal violet cell survival assay, we detected a significant loss of cell viability at sTRAIL concentrations as low as 0.01 ng/ml and again, the addition of MPs did not affect this activity (Fig. 5e). The BHM-MSC.sTRAIL-induced apoptosis was corroborated in two additional cancer cell lines (Additional file 3: Fig. S3).

In addition, we mixed MSC.sTRAIL and BHM-MSC.sTRAIL with MDA-MB-231 cells to mimic therapeutic MSCs being guided to tumours, infiltrating them and delivering their therapeutic payload (in this case sTRAIL). In these mixing experiments we detected no difference in the induced cell death (~ 50%) in MDA-MB-231 cells when treated with MSC.sTRAIL or BHM-MSC.sTRAIL (Fig. 5f, g). In contrast, no cell death could be detected when the breast cancer cells were mixed with control MSC.Luc and BHM-MSC.Luc (Fig. 5f, g). These results show BHM-MSC.sTRAIL are able to trigger cancer cell death in a context similar to what this cell might encounter in vivo, and that the transformation of MSCs into BHM-MSCs does not impede this function.

### Loading with MPs does not reduce the migration potential of MSCs

**Fig. 6:**
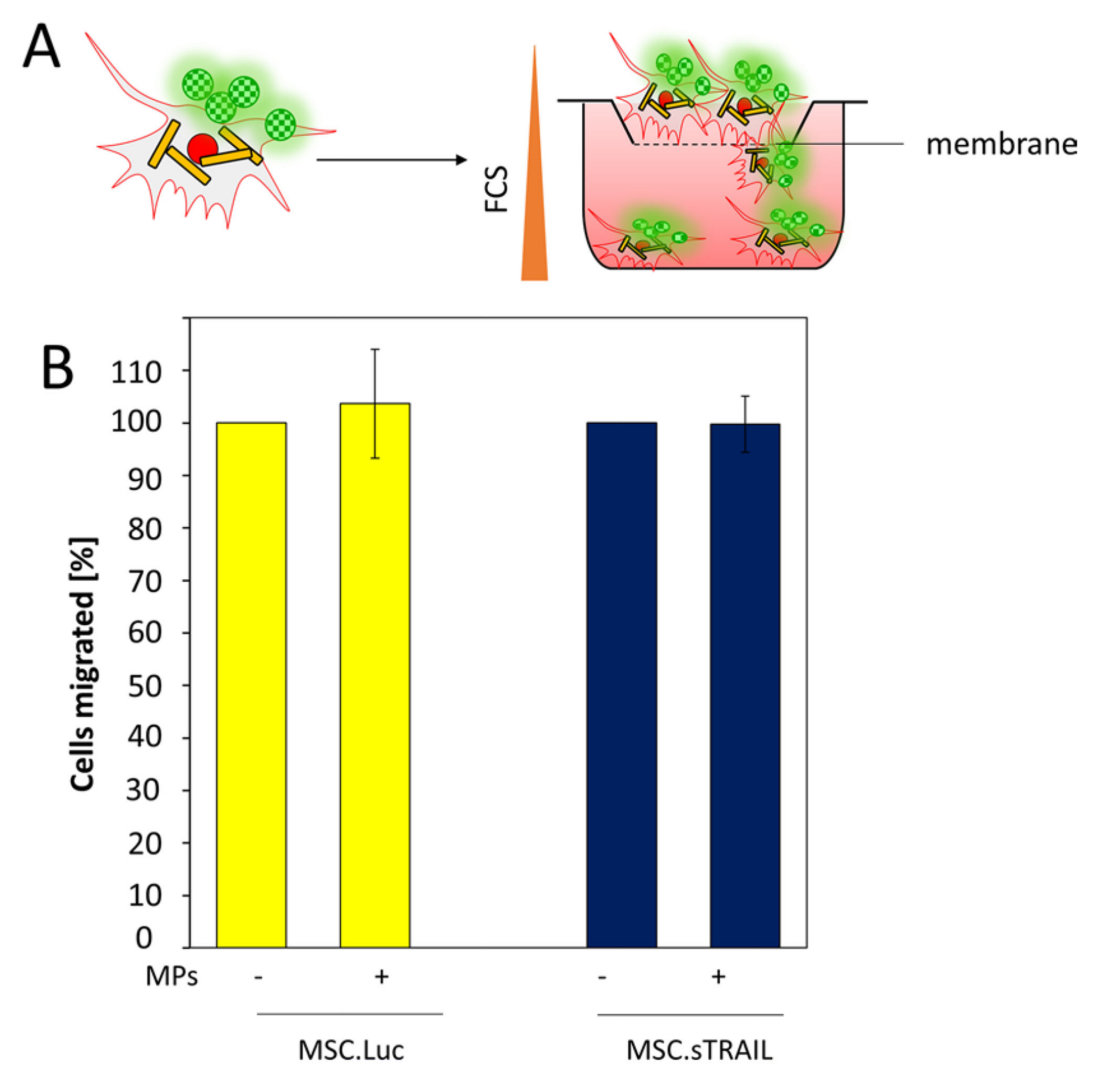
sTRAIL expression does not affect the migratory capacity of BHM-MSCs. **A** Schematic overview of the transwell migration assay. **B** MSCs transduced with adenoviral vectors expressing luciferase (MSC.Luc) or sTRAIL (MSC.sTRAIL), with (+ MPs) or without MPs (– MPs) were analysed for their migratory activity. The number of cells which crossed the membrane in the unloaded MSC samples were set to 100. For each group, cells from six transwells were quantified. Data are plotted as mean ± SEM

In order to use MSCs as BHMs they have to retain their biological migratory activity. To test this, we used BHM-MSCs and measured their ability to migrate across a 8 μm polycarbonate membrane (Fig. 6a). When we compared the BHM-MSCs to unloaded MSCs we found no difference in the number of cells that migrated through the membrane. These results demonstrate that MPs do not interfere with the migration potential of MSCs. Next, we used MSC.Luc, MSC.sTRAIL (Fig. 6b) and MSC.sPD1HAC (Additional file 4: Fig. S4) and compared them to their BHM counterparts. Again, no difference between the different cells and BHMs could be observed. This means that the generation of genetically engineered BHMs does not interfere with their biological motility. Thus, it is possible to use MSCs as a building block for BHMs in which their migratory activity will be responsible for the final part of the delivery route to target tissues such as tumours.

## Discussion

We prepared magnetically actuated stem cell-based BHMs by internalisation of MPs by MSCs. The goal of this work was to generate therapeutically active MSCs that can be guided by rotating magnetic fields. Rotating magnetic fields have an advantage over gradient-based controls in that they require lower magnetic field intensities, making them more suitable for scaling up and adapting to human applications (Hoshiar et al. 2020; Jarvis et al. 2022). Our results suggest that BHM-MSCs can be effectively steered and their direction controlled, while maintaining a constant dispersion over time, even at different intensities and MP concentrations. Thus, by using magnetic guidance, BHM-MSCs can potentially be delivered in a microrobot swarm and precisely directed to specific locations in the body. This targeted approach could reduce the required dosage of MSCs per treatment schedule and lower the risk of adverse side effects.

Intravenously administered MSCs are known to start accumulating in the lungs shortly after injection but do not persist in the tissue for more than 24 h in significant numbers (Kidd et al. 2009; Lee et al. 2009). Biodistribution analyses at longer timepoints that are more relevant for achieving therapeutic effects revealed that MSCs are present in lymph nodes, spleen, liver and bone marrow as well as in very small numbers in the lungs (Lee et al. 2009; Albarenque et al. 2011). In similar studies using tumour bearing animals, MSCs were also found in tumours without affecting the overall biodistribution (Albarenque et al. 2011). Thus, the ability to better guide therapeutic MSCs to their target tissue(s), like the primary tumour and secondary metastatic lesions, would make more efficient use of the injected cells and would limit potential side effects.

Most importantly, magnetic guidance could avoid MSCs getting trapped in the lungs, thereby increasing their availability for therapeutic purposes in other tissues. While magnetic guidance in this context will achieve the broad tissue targeting, the biological activity of MSCs is required for the last leg of the delivery. For this it is important that MSCs loaded with MPs do not lose their migratory activity in order to be able to complete the final stage of migration within their target tissues. Therefore, we measured the migratory potential of our therapeutic BHM-MSCs and compared them to normal MSCs. As we found no difference between the migration activity between the two groups, we concluded that BHM-MSCs do not lose their biological ability to infiltrate tissues, which is crucial for the completion of the delivery following the broad guidance by magnetic force. This will ensure that our therapeutic BHM-MSCs will complete their journey to their target tissues on their own devices.

While the generation of BHM-MSCs has been described before (Go et al. 2020), very little is known about the compatibility of the loading with MPs and the functional expression of therapeutic genes by MSCs. MSCs without such therapeutic payload can be useful in many settings but the additional genetic modification of the BHM-MSCs offers an even wider scope of therapeutic applications and benefits, in particular in cancer treatments (Go et al. 2020). In this context, several therapeutic genes have been tested (Sage et al. 2016; Mohr and Zwacka 2018), of which some progressed to clinical trials (Mohr and Zwacka 2018; Sage et al. 2016; Levy et al. 2020). One example of this is the TACTICAL study in which MSCs expressing TRAIL are used for the treatment of lung cancer. While in this approach the initial infiltration of MSCs into the lungs is desired, in other cancer types targeting of other tissues will be required, e.g. metastatic lesions in the liver. In our present study we used expression of the reporter gene luciferase as well as two therapeutic genes, sTRAIL and sPD1HAC. In all three cases we did not detect a negative impact of the MPs on transgene expression.

## Conclusion

BHM-MSCs can be produced with concentrations of MPs that do not negatively affect their viability, motility as well as therapeutic transgene expression and function. At the same time, these MP concentrations allow control and guidance over the BHM-MSCs in a rotating magnetic field. Hence, it is technically possible to manufacture therapeutic BHM-MSCs to be tested in experimental cancer therapy models next. In order to further optimise the generation of BHM-MSCs, the biophysical characteristics of the MPs, such as their hydrodynamic size, have to be considered in both modelling and experimental approaches.

## Methods

### Materials

All chemicals, unless otherwise stated, were purchased from Thermo Fisher Scientific (MA, USA). Tissue culture materials and plastics were bought from Sarstedt (Nuembrecht, Germany). The BCL_XL_-inhibitor (A1155453) was purchased from Selleckchem (TX, USA).

### Cell culture

The breast cancer cell line, MDA-MB-231 (ATCC, VA, USA) was cultured in DMEM (Lonza, Switzerland). The multiple myeloma cell line MM1R (ATCC, VA, USA) was cultured in RPMI (Lonza, Switzerland). The colorectal cancer cell line RKO (ATCC, VA, USA) was cultured in McCoys (Lonza, Switzerland). All media were supplemented with 10% foetal bovine serum (FBS) (Thermo Fisher Scientific, MA, USA), 100 U/ml penicillin and 100 μg/ml streptomycin. Human MSCs were from Thermo Fisher Scientific (MA, USA) and cultured in MesenPro RS medium (Thermo Fisher Scientific, MA, USA).

### Cell labelling with MPs

Human MSCs were labelled with 1 μm MPs (SiMAG-Silanol, Chemicell, Germany) at different concentrations. The SiMAG-Silanol particles consist of an unmodified silica surface. The coating material of SiMAG particles is amorphous SiO_2_ with terminal negatively charged silanol groups (Si–OH) and iron oxide cores (5–20 nm) (Park et al. 2023). The iron oxide cores consist of maghemite (Fe_2_O_3_). In the MPs, several iron oxide cores with different core diameters are embedded in a SiO_2_ matrix, with a total diameter of 1 μm. The hydrodynamic particle diameter of 1 μm was determined by Dynamic Light Scattering. According to Chemicell, the magnetic properties of the MPs are: 58 Ms [emu/g], 2.4 Hc [Oe], Mr/Ms 0.0086. Further details about the magnetic properties of the MPs have been reported by Oduwole et al. (Oduwole et al. 2016). They used a linear interpolation within the applied flux density range (± 50 mT) to estimate the magnetic susceptibility of the bead (χ = 1.3) at 11 mT (Oduwole et al. 2016). For the loading, adherent cell populations were starved for 6 h before MPs (0.6–10 μg/ml) were applied in serum-free medium for 24 h. The next day, the cells were thoroughly washed 5 times with phosphate-buffered saline (PBS) in order to remove excess particles.

### Measurement of agglomeration of MPs

To investigate the colloidal stability in different media, nanoparticles were incubated at different concentrations in DMEM with 10%, 2% or 0% FBS or PBS with 2% FBS overnight under cell culture conditions. The next day, 100 μl supernatant (SN) of each sample, as well as 100 μl of remaining fluid (SM) with resuspended MPs, was transferred to separate wells of a 96-well plate. The optical density of the samples was measured at 320 nm as described by Boosz et al. (Boosz et al. 2021).

### Prussian blue staining

Cells were fixed with 4% paraformaldehyde (PFA) for 15 min and subsequently rinsed with deionised water. Freshly prepared, 20% aqueous solution of hydrochloric acid and 10% aqueous solution of potassium ferrocyanide were mixed in equal parts and added to the cells for 20 min. The staining solution was removed, and the cells were washed three times with deionised water. The stained cells were imaged in 200 μl deionised water using an Evos M5000 microscope (Thermo Fisher Scientific, MA, USA).

### Cell viability assay

Impact on membrane integrity was assessed using a live/dead cell imaging kit (Thermo Fisher Scientific, MA, USA) according to the manufacturer’s instructions. As a positive control for the dead stain, cells were killed and fixed with ethanol. The stained cells were imaged using an Evos M5000 microscope (Thermo Fisher Scientific, MA, USA).

### DNA-hypodiploidy assays

Apoptosis was measured according to Nicoletti et al. (Nicoletti et al. 1991). Briefly, 24 h post-treatment, cells were harvested and washed once with PBS. Pelleted cells were resuspended in hypotonic fluorochrome solution containing 50 μg/ml propidium iodide, 0.1% sodium citrate, and 0.1% Triton-X100. After incubation at 4 °C for 2 h, cells were analysed by flow cytometry.

### ELISA

Cellular supernatants were removed and cleared by centrifugation. TRAIL and PD1 protein levels were determined by DuoSet ELISA (Biotechne, MN, USA), according to manufacturer’s instructions.

### Adenoviral transduction

Adenoviral vectors expressing luciferase (Ad.Luc) and sPD1HAC (Ad.sPD1HAC) (Maute et al. 2015; Pascolutti et al. 2016) were purchased from Vector Biolabs (PA, USA). Ad.sTRAIL was generated as previously described (Mohr et al. 2019). For adenoviral transduction, the normal growth medium was changed to medium supplemented with 2% FBS. The cells were transduced with virus at 200 pfu/cell for 6 h and then washed.

### Surface marker stain

The surface marker expression was measured by incubating MSCs with PE-conjugated mouse anti-human antibodies for CD44 (BJ18), CD90 (5E10), CD105 (43A3) or APC-conjugated mouse anti-human antibodies for CD45 (HI30) followed by flow cytometric analysis. PE-conjugated mouse IgG1κ and APC-conjugated mouse IgG1κ served as isotype controls. All antibodies were purchased from Biolegend (CA, USA).

### Migration assay

Migration of BHM-MSCs and MSCs was measured with a Cell Migration/Chemotaxis Assay Kit (Abcam UK), according to the manufacturer’s instructions. Briefly, transgene expressing BHM-MSCs were cultured in serum-free medium for 24 h before being trypsinised and resuspended in serum-free medium. BHM-MSCs were plated in the wells containing the insert chambers at a density of 50,000 cells per well in serum-free medium. 10% FBS-containing medium was then added to the lower chamber to serve as a chemoattractant. Cells were allowed 24 h to migrate across the 8 μm polycarbonate membrane. Cell migration was analysed by reading fluorescence (Ex/Em = 530/590 nm) in a plate reader.

### Luciferase assay

Luciferase expressing BHM-MSCs were washed five times with PBS before the addition of 1 × passive lysis buffer (Promega, WI, USA). The culture vessel was gently rocked for 15 min at room temperature before the lysate was transferred to a microcentrifuge. 5 μl of the lysate was added into a black 96-well plate with a clear bottom (Corning, NY, USA) containing Luciferase Assay Reagent II (Promega, WI, USA). Luminescence was measured in a luminometer.

### Crystal violet staining

Cells were seeded at a density of 1 × 10^4^ cells in a 24-well plate. After 24 h the cells were treated with different concentrations of sTRAIL derived from MSC.sTRAIL. After 7 days, resulting colonies were fixed in 2% PFA and visualised with a 0.04% crystal violet solution. The crystal violet dye was solubilised in methanol and the optical density of each well was measured at 570 nm. The average OD_570_ of non-stimulated cells was set to 100%.

### Magnetic actuation

The experimental setup consists of an MFG100 system (Magnebotix, Switzerland), optical camera (Basler, puA1280-54uc). The magnetic field generator can generate a controlled 3D magnetic field (< 20 mT). Different field frequencies (< 20 Hz) and intensities (< 15 mT) were used to steer BHM-MSCs and study the steering performance. The MATLAB image processing toolbox was used to characterise the biohybrid microswarm performance. In all experiments, the same region of interest was considered for image processing.

### Statistical analysis

Experimental values are expressed as mean value ± standard error of the mean (SEM). For significance analyses, analysis of variance (ANOVA) between groups was used and *p ≤ 0.05 was considered significant, **p ≤ 0.01 as highly significant and ***p ≤ 0.001 as extremely significant. Experiments were performed three times unless otherwise stated.

## Supplementary Material

Supp 1

Supp 2

Supp 3

Supp 4

Supp 5

Supplementary Material

## Data Availability

Not applicable.
