# Supplementary figures and images for: Generation of magnetic biohybrid microrobots based on MSC.sTRAIL for targeted stem cell delivery and treatment of cancer
Source: Cancer Nanotechnol. Author manuscript; Available in PMC 2023 Oct 20. (PMC7615227; doi:10.1186/s12645-023-00203-9)

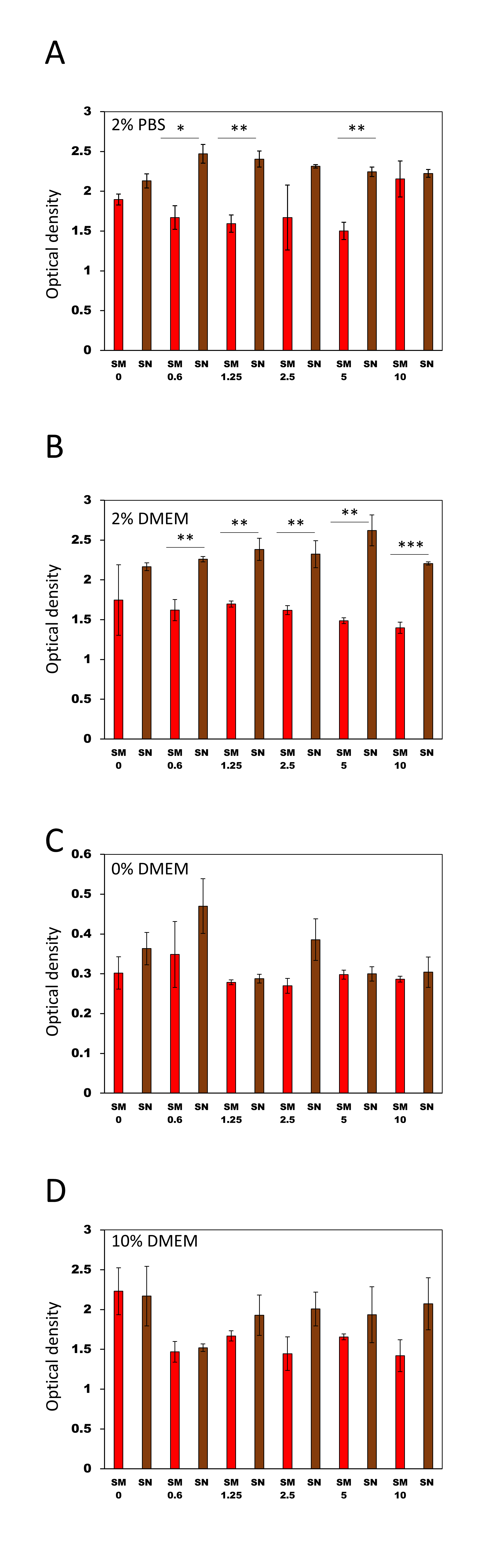

Supplement: Supp 1 [file EMS188685-supplement-Supp_1.tif]

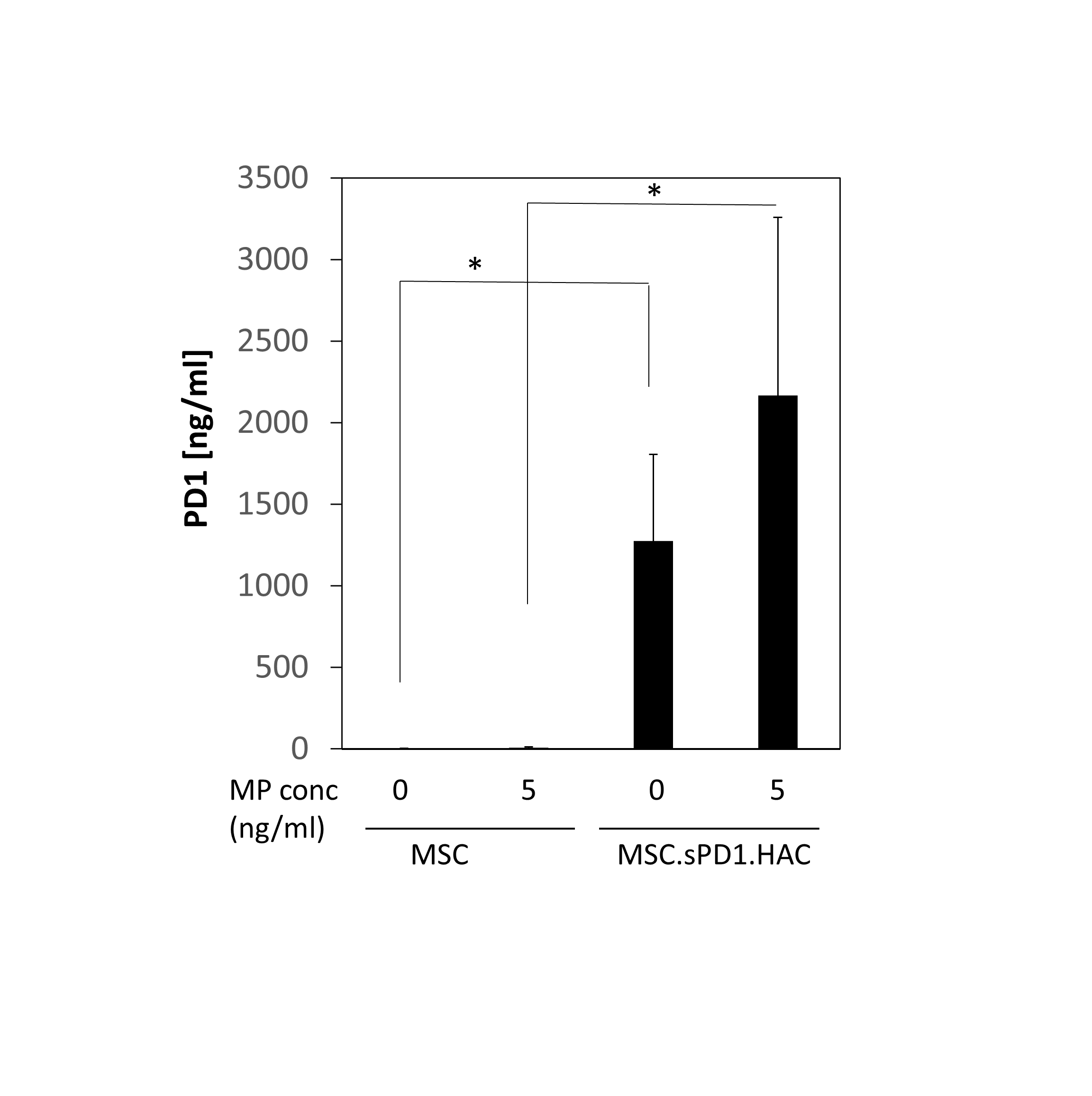

Supplement: Supp 2 [file EMS188685-supplement-Supp_2.tif]

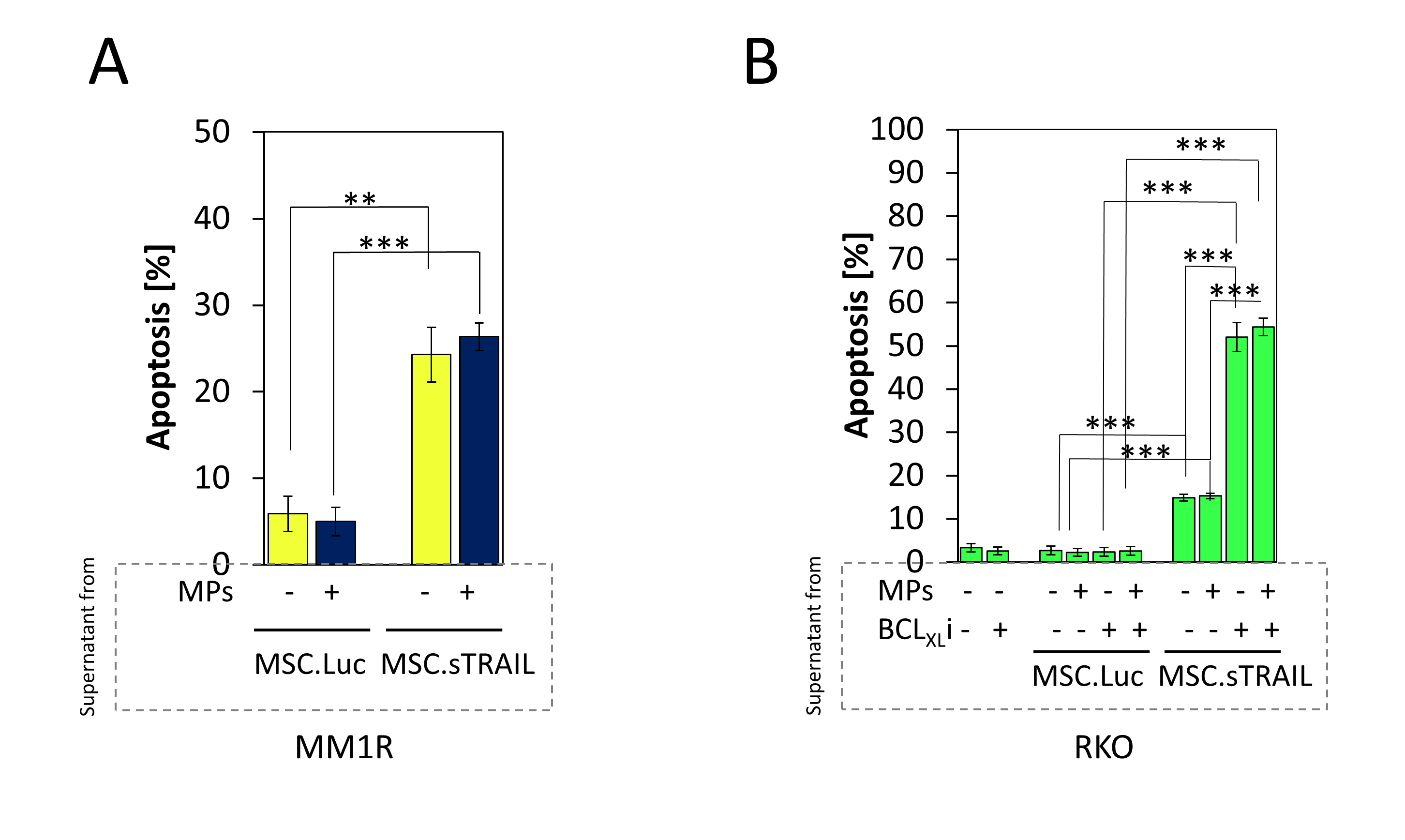

Supplement: Supp 3 [file EMS188685-supplement-Supp_3.tif]

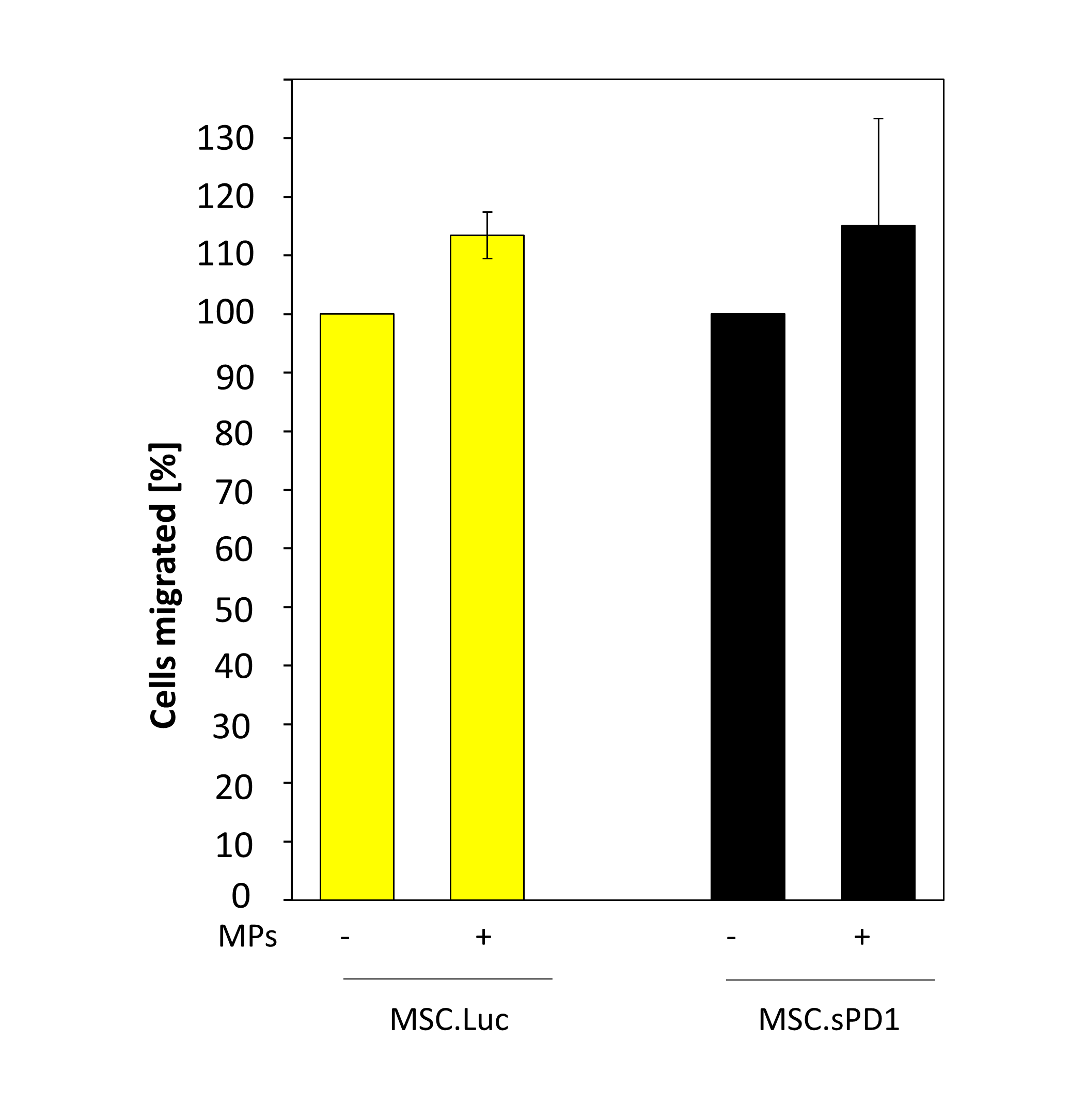

Supplement: Supp 4 [file EMS188685-supplement-Supp_4.tif]
