# Supplementary material for: Generation of magnetic biohybrid microrobots based on MSC.sTRAIL for targeted stem cell delivery and treatment of cancer
Source: Cancer Nanotechnol. Author manuscript; Available in PMC 2023 Oct 20. (PMC7615227; doi:10.1186/s12645-023-00203-9)
Supplement: Supplementary Material [file EMS188685-supplement-Supplementary_Material.pdf]

Supplementary Information

The online version contains supplementary material available at https://doi.org/10.1186/s12645-023-00203-9.

Additional file 1. Fig. S1: Colloidal stability of MPs in different media. MPs were diluted in different media at concentrations ranging from 0 to 10 ng/ml and incubated overnight under cell culture conditions. Optical density was measured at 320 nm of the supernatantand the remaining resuspended samples. **A** MPs diluted in PBS supplemented with 2% FBS. **B** MPs analysed in DMEM containing 2% FBS. **C** DMEM without FBSand **D** DMEM supplemented with 10% FBS; data are plotted as mean ± SEM.

Additional file 2. Fig. S2: BHM-MSCs express sPD1HAC. Untransduced MSCsor MSCs transduced with an adenoviral vector expressing sPD1HAC, withor without MPs; Data are plotted as mean ± SEM.

Additional file 3. Fig. S3: Multiple myeloma and colorectal cancer cells are killed by sTRAIL produced by BHM-MSC. sTRAIL. **A** MM1R multiple myeloma cells treated with supernatants from MSCs transduced with adenoviral vectors expressing luciferaseor sTRAIL, withor without MPs. Apoptosis was measured by DNA-hypodiploidy assay and flow cytometry; Data are plotted as mean ± S.E.M.. **B** RKO colorectal cancer cells treated with supernatants from MSCs transduced with adenoviral vectors expressing luciferaseor sTRAIL, withor without MPs. A BCLXL-inhibitor was used at a concentration of 2 μM to sensitise RKO cells to apoptosis. Untreated MSCs and BCLXL-inhibitor treated cells served as controls. Apoptosis was measured by DNA-hypodiploidy assay and flow cytometry; Data are plotted as mean ± SEM..

Additional file 4. Fig. S4: sPD1HAC expression does not affect the migratory capacity of BHM-MSCs. MSCs transduced with adenoviral vectors expressing luciferaseor sPD1HAC, withor without MPswere analysed for their migratory activity. The number of cells which crossed the membrane in the unloaded samples were set to 100%. For each group, cells from three transwells were quantified. Data are plotted as mean ± SEM.

Additional file 5. Moving BHM-MSCs under a rotating magnetic actuation.
